# Neurogranin Targets Calmodulin and Lowers the Threshold for the Induction of Long-Term Potentiation

**DOI:** 10.1371/journal.pone.0041275

**Published:** 2012-07-25

**Authors:** Ling Zhong, Nashaat Z. Gerges

**Affiliations:** Department of Cell Biology, Neurobiology, and Anatomy, Medical College of Wisconsin, Milwaukee, Wisconsin, United States of America; University of Michigan, United States of America

## Abstract

Calcium entry and the subsequent activation of CaMKII trigger synaptic plasticity in many brain regions. The induction of long-term potentiation (LTP) in the CA1 region of the hippocampus requires a relatively high amount of calcium-calmodulin. This requirement is usually explained, based on *in vitro* and theoretical studies, by the low affinity of CaMKII for calmodulin. An untested hypothesis, however, is that calmodulin is not randomly distributed within the spine and its targeting within the spine regulates LTP. We have previously shown that overexpression of neurogranin enhances synaptic strength in a calmodulin-dependent manner. Here, using post-embedding immunogold labeling, we show that calmodulin is not randomly distributed, but spatially organized in the spine. Moreover, neurogranin regulates calmodulin distribution such that its overexpression concentrates calmodulin closer to the plasma membrane, where a high level of CaMKII immunogold labeling is also found. Interestingly, the targeting of calmodulin by neurogranin results in lowering the threshold for LTP induction. These findings highlight the significance of calmodulin targeting within the spine in synaptic plasticity.

## Introduction

In CA1 region of the hippocampus, long-lasting changes in synaptic efficacy depend on neuronal activity and are widely accepted as the cellular correlates of learning and memory formation [1], [2], [3]. A well-characterized form of synaptic plasticity is long-term potentiation (LTP). LTP induction requires the activation of NMDA receptors and a relatively large increase (a few micromolars) in Ca^2+^ concentration within dendritic spines. This increase in local Ca^2+^ over a short period of time (a few seconds) causes a conformational change in calmodulin (CaM) allowing it to activate Ca^2+^/CaM-dependent protein kinase II (CaMKII), which mediates AMPA receptor (AMPAR) delivery to synapses. Interestingly, a small increase in postsynaptic Ca^2+^ causes CaM to activate calcineurin, resulting in the expression of long-term depression (LTD). Thus, through its activation of two different prominent pathways within the same spine, CaM can lead to either LTP or LTD.

This intriguing property of CaM to differentially activate these opposing targets within the dendritic spines has been explained by the differential affinity of these targets for CaM. For example, the requirement of high Ca^2+^ levels for LTP induction is usually explained by the need to overcome the low affinity of CaMKII for CaM [4], [5], [6]. This explanation, however, does not take into account the complex organization of the dendritic spine and the spatial distributions of CaM and CaMKII within the spine.

Neurogranin (Ng) is a postsynaptic protein whose main known function is to bind the Ca^2+^-free form of CaM (apo-CaM) [7], [8], [9]. Two main views exist regarding the relevance of such binding. According to one view, Ng sequesters CaM and thereby inhibits its ability to activate subsequent targets [10], [11]. The other view, however, is that Ng concentrates and/or targets CaM within dendritic spines to facilitate Ca^2+^/CaM-mediated signaling [6], [12], [13]. In support of the latter view, we have shown that overexpression of Ng increases CaMKII activation and enhances synaptic strength [14]. Importantly, Ng mutants that are incapable of binding to CaM, or those that constitutively bind to CaM even in high Ca^2+^ levels [14], are incapable of enhancing synaptic strength. Collectively, the physiological relevance of Ng is centered on its ability to bind and regulate CaM. One possibility is that increasing synaptic Ng enhances synaptic strength mainly due to a generalized increase in CaM levels within the spine. The generalized increase of CaM *per se*, however, does not enhance synaptic strength [14]. As a result, Ng-mediated enhancement of synaptic strength may require CaM targeting within the spine [14].

Here, we show that CaM distribution in dendritic spines of CA1 hippocampal neurons is not random and that it can be preferentially targeted closer to the plasma membrane by Ng. Our results also revealed that dendritic CaMKII has a lateral distribution similar to that of CaM, and targeting of CaM by Ng lowers the threshold for LTP induction. These findings suggest that the spatial targeting of CaM and the localization of CaMKII play an important role in the induction of LTP at hippocampal CA1 synapses.

## Materials and Methods

### Animals and Hippocampal Slice Preparation

Young Sprague-Dawley rats (postnatal day 5 or 6) were purchased from Charles River Laboratories (Portage, MI, USA) and maintained on a 12 h light/dark cycle (lights off at 6:00 P.M.). Organotypic hippocampal slices were prepared as described previously [15]. All biosafety procedures and animal care protocols described here were approved by the Medical College of Wisconsin Institutional Animal Care and Use Committee and were performed in strict accordance with the Guidelines for Care and Use of Laboratory Animals of the National Institutes of Health.

### DNA Constructs and Expression

Ng was cloned by PCR from a commercial rat brain cDNA (Clontech, CA, USA). GFP-Ng was made with pEGFP plasmid and re-cloned into pSinRep5 (Invitrogen, NY, USA) for virus preparation as described [14]. After 5–7 days in culture, GFP-Ng was delivered into the slices using the Sindbis virus expression system, which is a replication-deficient, low-toxicity and neuron-specific system [16].

### Post-embedding Immunogold Electron Microscopy

Organotypic hippocampal slices were fixed and processed for osmium-free post-embedding immunogold labeling essentially described earlier [17]. Briefly, CA1 region was carefully removed from the hippocampal slice and fixed with 0.1% picric acid, 1% paraformaldehyde, and 2.5% glutaraldehyde in 0.1 M phosphate buffer (pH 7.3) for 2 h at 4°C. After fixation, tissues were washed in 0.1 M maleate buffer (pH 6.0), treated with 1% tannic acid, 1% uranyl acetate, and 0.5% platinum chloride, followed by dehydration through a series of ethanol solutions. Tissues were then embedded in epoxy resins, sectioned and stained with 1% toluidine blue and 1% borax. CaM was labeled with an anti-CaM antibody (Epitomics, CA, USA) and an anti-rabbit antibody coupled to 10-nm gold particles (Electron Microscopy Sciences, PA, USA). CaMKII was labeled with an anti-CaMKIIα antibody (a generous gift from Dr. Johannes Hell, University of California-Davis) and an anti-mouse antibody coupled to 15-nm gold particles (Electron Microscopy Sciences). Electron micrographs were obtained with a JOEL EM-2100 transmission electron microscope and an Orius SC 1000 CCD camera (JOEL, MA, USA).

### Electrophysiology

Synaptic responses in organotypic slice cultures were evoked with two bipolar electrodes (FHC, ME, USA) placed on the Schaffer collateral fibers between 300 and 500 mm of the recorded cells. The recording chamber was perfused with 119 mM NaCl, 2.5 mM KCl, 4 mM CaCl_2_, 4 mM MgCl_2_, 26 mM NaHCO_3_, 1 mM NaH_2_PO_4_, 11 mM glucose, 0.1 mM picrotoxin and 2 µM 2-chloroadenosine, at pH 7.4, and gassed with 5% CO_2_, 95% O_2_. 0.1 mM DL-2-amino-5-phosphonopentanoate (AP5) (R&D Systems, MN, USA) was present in the bath solution. Patch recording pipettes (3–6 M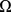) were filled with 115 mM cesium methanesulfonate, 20 mM CsCl, 10 mM HEPES, 2.5 mM MgCl_2_, 4 mM Na_2_ATP, 0.4 mM Na_3_GTP, 10 mM sodium phosphocreatine and 0.6 mM EGTA, at pH 7.25. LTP was induced by pairing 3 Hz presynaptic stimulation (300 pulses) with -20 mV postsynaptic depolarization. Voltage-clamp whole-cell recordings were acquired with a Multiclamp 700A amplifier (Axon Instruments, CA, USA).

### Statistical Analysis

Comparison of normalized average steady-state AMPA receptor-mediated responses between control uninfected neurons and Ng-infected neurons were carried out using unpaired *t*-tests. Comparison of cumulative distributions was carried out with the Kolmogorov-Smirnov test. Comparison of the distribution of CaM within the different sub-compartments, within the synapse, between control and Ng-infected conditions was achieved using Chi-squared test. Values were considered significantly different if *p* ≤ 0.05, and marked with an asterisk. Error bars represent standard error of the mean in all figures.

## Results

### CaM is not Randomly Distributed within Dendritic Spine

**Figure 1:**
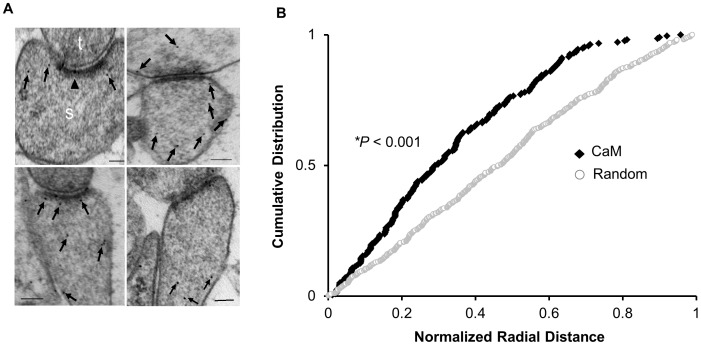
CaM is not randomly distributed within the synapse. (**A**) Representative images of immunogold-EM for endogenous CaM in dendritic spines of CA1 hippocampal neurons. The postsynaptic compartment (*s*) is recognized by the appearance of the prominent postsynaptic density (black arrowhead) and well-defined plasma membrane. *t*, presynaptic terminal. Arrows indicate anti-CaM immunogold particles. Scale bar, 100 nm. (**B**) Cumulative probabilities of normalized radial distance. The distance of each gold particle from the plasma membrane (within the spine) was normalized (x axis) to the corresponding radius of the spine measured through that particle. The distribution of CaM within the spine is significantly different from a theoretical random distribution (n = 214, *p*<0.001).

As mentioned earlier, overexpression of CaM, unlike Ng, was incapable of enhancing synaptic strength [14]. The theory that a high CaM concentration is required for CaMKII activation because of its low affinity for CaM cannot fully explain the lack of sufficient CaMKII activation to produce LTP-like changes when CaM is overexpressed. Thus, CaM localization in the spine may have a direct impact on the subsequent signaling cascade mediated by this enzyme. To test this possibility, we first wished to understand the precise ultrastructural localization of endogenous CaM within dendritic spines. To do so, we used post-embedding anti-CaM immunogold labeling on synaptic region of the CA1 stratum radiatum. Most of the synapses examined contained CaM labeling, with labeling in both the pre- and the postsynaptic compartments (56% presynaptic and 44% postsynaptic). To quantitatively assess the ultrastructural localization of CaM postsynaptically, we used a method of immunogold-electron microscopy (EM) similar to the one described previously [14], [18]. Briefly, the shortest distance of each gold particle to the plasma membrane was measured and then normalized to the radius of the spine. As shown in Figure 1B, CaM localization in the spine is significantly different from a random distribution (*p*<0.001). Furthermore, CaM is preferentially localized near the plasma membrane. These results support a model in which CaM is spatially targeted and that its localization within dendritic spine may influence synaptic function.

### Ng Targets and Relocates CaM Closer to Plasma Membrane

**Figure 2:**
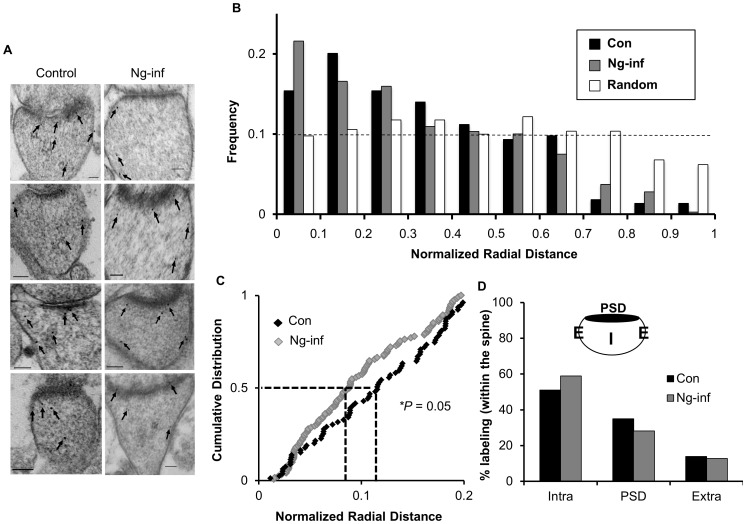
Ng targets and relocates dendritic CaM closer to plasma membrane of the postsynaptic compartment. (**A**) Representative images of immunogold-EM for CaM in control and Ng-overexpressing neurons. Scale bar, 100 nm. (**B**) Frequency histograms of CaM (n = 214) in control neurons and Ng-inf (n = 319) neurons. Normalized radial distance was measured the same way as described in Figure 1. Note the increased fraction of CaM at 0.1 interval in Ng-expressed condition. (**C**) Cumulative probabilities of normalized radial distance of CaM particles located within 0.2 of the normalized radial distance, where Ng was found to be concentrated in our previous study. Ng targeted CaM closer to the plasma membrane (*p* = 0.05). (**D**) Ng did not change the global distribution of CaM in the spine (PSD: postsynaptic density; E: extrasynaptic membrane; I: intraspine). The *p-*value for comparing cumulative distribution was calculated using Kolmogorov-Smirnov test. The *p-*value for comparing global CaM distribution in different sub-compartments was calculated using Chi-squared test.

One of the most abundant CaM-binding proteins in the CA1 region of the hippocampus is Ng, which is concentrated in the spine [14], [19], [20]. Similar to the CaM distribution described here, endogenous Ng was found to be preferentially localized near the plasma membrane of the postsynaptic compartment [14], suggesting that it may target CaM within the spine. To investigate whether Ng influences CaM localization, we performed immunogold-EM labeling of CaM in tissues overexpressing Ng. Since overexpression of Ng results in NMDAR-dependent AMPAR insertion, synaptic potentiation and LTP occlusion [14], we incubated the slices with the NMDAR antagonist AP5 during Ng expression. This configuration allows us to test the effects of Ng on CaM distribution without the potential secondary effects that would stem from the LTP-like changes mediated by Ng overexpression [14]. Representative micrographs in Figure 2A highlight the abundance of CaM immunogold labeling near the plasma membrane in Ng-infected neurons. Quantitative examination of CaM distribution shows that Ng overexpression increased the fraction of CaM that is closer to the plasma membrane (Fig. 2B). Importantly, cumulative distribution of gold particles revealed that Ng overexpression significantly shifted CaM closer to the plasma membrane (Fig. 2C). Despite this localized targeting of CaM by Ng, it is important to note that Ng overexpression did not significantly change the global distribution of CaM (using Chi-squared test) in the different sub-compartments within the spine (Fig. 2D). These data indicate that Ng targets a pool of CaM within the dendiritc spines close to the plasma membrane.

### Ultrastructural Localization of CaMKII

**Figure 3:**
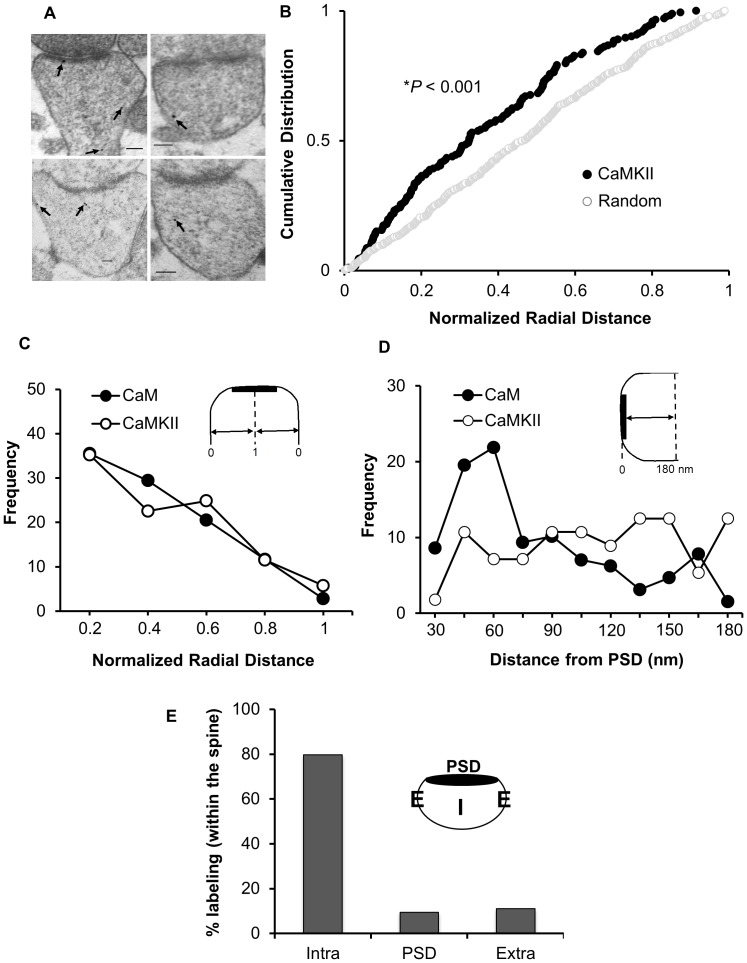
CaMKII lateral distribution correlates with that of CaM within the spine. (**A**) Representative images of immunogold-EM for endogenous CaMKII in dendritic spines of CA1 hippocampal neurons. Arrows indicate anti-CaMKII immunogold particles. Scale bar, 100 nm. (**B**) Cumulative probabilities of normalized radial distance. The distance of each gold particle from the plasma membrane (within the spine) was normalized (x axis) to the corresponding radius of the spine measured through that particle. CaMKII distribution within the spine is significantly different from a theoretical random distribution (n = 173, *p*<0.001). (**C**) Comparison of CaMKII and CaM distribution using cumulative probabilities of normalized radial distance. CaMKII and CaM show similar patterns of lateral distribution. The highest fraction of labeling for both occurs within 0.2 of the radial distance. (**D**) Vertical distribution of CaMKII and CaM with respect to PSD. The absolute distance between each gold particle to the PSD was measured. (**E**) Distribution of CaMKII between the different sub-compartments within the spine.

As mentioned above, CaMKII requires CaM for its activation and is a key molecule in LTP induction. We thus wished to test whether there is any correlation between CaMKII and CaM localizations within the spine. To address this question, we carried out immunogold-EM similar to that described above using anti-αCaMKII antibody (Fig. 3A). As shown in Figure 3B, CaMKII distribution in the spine is not random. Instead, it showed a lateral localization similar to that of CaM (Fig. 3C). Interestingly, the vertical distributions are distinctly different between CaMKII and CaM (Fig. 3D). It is also worth noting that while there is a significant fraction of CaM immunolabeling at the postsynaptic density (PSD; Fig. 2D), CaMKII immunolabeling at the PSD is much less (Fig. 3E). Collectively, these data suggest that the CaM pool that is close to the extrasynaptic plasma membrane is more relevant than that at the PSD, with respect to CaMKII.

### CaM Targeting within the Spine Lowers LTP Threshold

**Figure 4:**
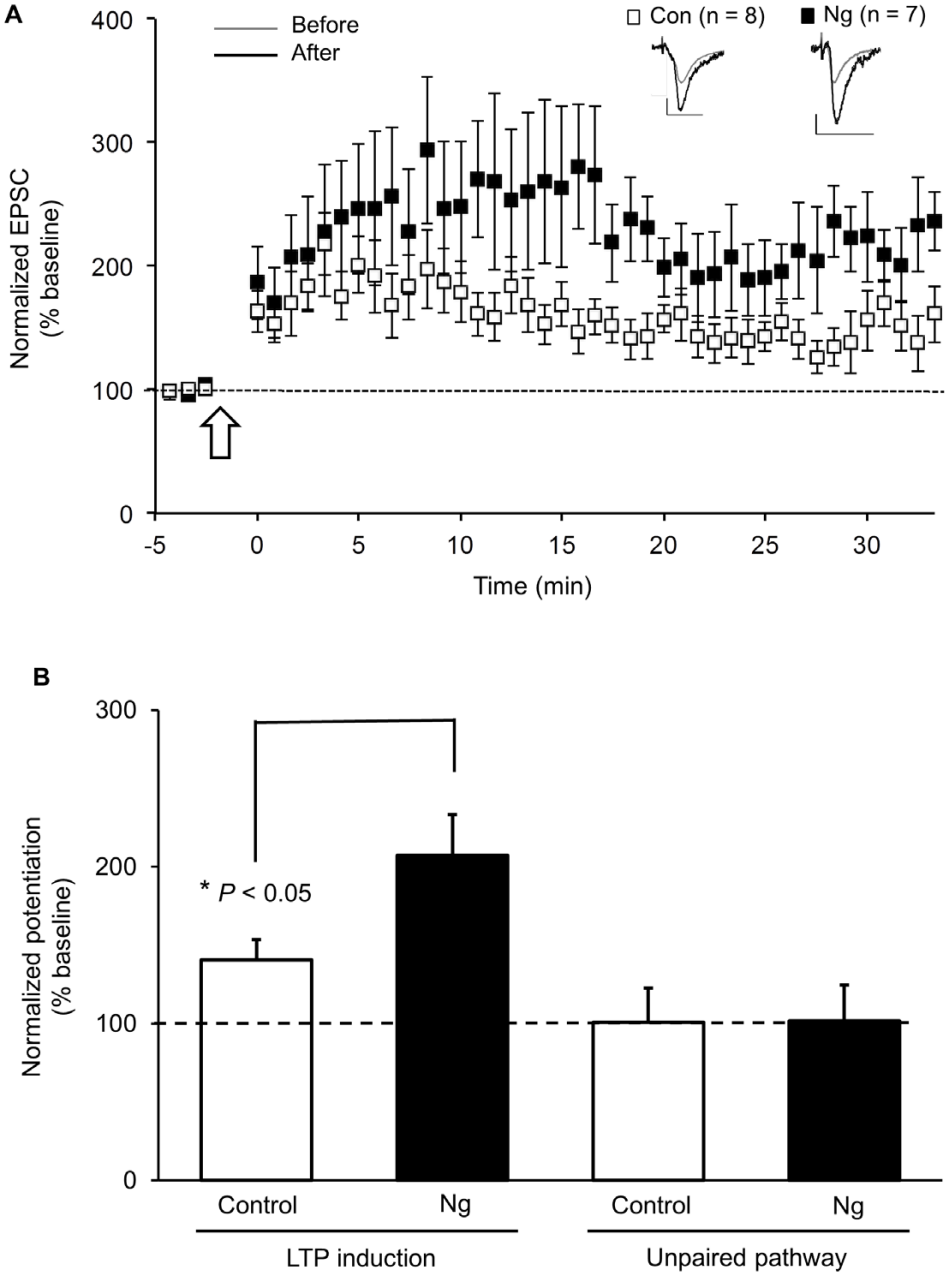
Ng lowers LTP threshold in CA1 hippocampal pyramidal neurons. (**A**) LTP was induced by pairing 3-Hz presynaptic stimulation (300 pulses) with −20 mV postsynaptic depolarization (indicated with an arrow) in uninfected neurons (open squares, *n* = 8) and neurons expressing GFP-Ng (black squares, *n* = 7). Inserts: sample traces of evoked AMPAR-mediated synaptic responses recorded at −60 mV before pairing (gray line) and 20 min after pairing (black line) from control or infected cells as indicated. Scale bars: 20 pA, 20 ms. (**B**) Normalized average steady-state AMPAR-mediated responses in paired (LTP pathway) and unpaired (control pathways) for uninfected neurons and those expressing GFP-Ng. Pairing significantly increased AMPAR-mediated responses in both groups. Neurons expressing GFP-Ng showed higher level of LTP than control neurons (*p*<0.05).

Ng-mediated targeting of CaM to plasma membrane, where CaMKII also showed preferential localization, supports the hypothesis that Ng targets CaM to favor the activation of CaMKII and increases spine sensitivity to local Ca^2+^. Therefore, we tested whether CaM targeting can lower the threshold needed for LTP. To achieve this goal, we overexpressed Ng overnight in organotypic hippocampal slices. Similar to the tissues processed for immunogold-EM, AP5 was added concomitantly with Ng overexpression to prevent the Ng-mediated potentiation overnight [14]. The traditional pairing induction protocol for whole-cell recordings (by pairing presynaptic stimulation (3 Hz, 1.5 min) with postsynaptic depolarization of 0 mV) produces a significant increase in local Ca^2+^ and can have a ceiling effect on LTP magnitude [14], [21], [22], [23], [24]. For this reason, we chose an induction protocol with less depolarization to decrease the magnitude of Ca^2+^ entry during the induction. Here, a presynaptic stimulation (3 Hz, 1.5 min) was paired with a reduced postsynaptic depolarization (at −20 mV). This protocol was able to induce a modest level of LTP in control, uninfected neurons (Fig. 4A). Neurons expressing Ng, however, were able to express robust LTP at a higher level than control uninfected neurons (Fig. 4B). This finding suggests that Ng-mediated CaM targeting increases spine sensitivity to Ca^2+^ and lowers the threshold for LTP induction.

## Discussion

The requirement of a relatively high level of Ca^2+^ for LTP induction is usually explained by the low affinity of CaM to CaMKII, whose activation is required for LTP expression. In this study, we explored the hypothesis that CaM localization within the dendritic spine regulates LTP induction. First, we demonstrated that CaM exhibits a non-random distribution in the spine where a significantly greater fraction of CaM is localized close to the plasma membrane than would be predicted from a random distribution. We also found that the lateral distribution of CaM resembles that of CaMKII. Interestingly, increasing Ng expression in CA1 hippocampal neurons concentrates CaM closer to the extrasynaptic plasma membrane and results in lowering the threshold of LTP induction. These results reveal a novel mechanism by which CaM can control plasticity within dendritic spines.

CaM is a regulatory protein that modulates the activity of many signaling molecules in the cell. Intriguingly, some of these targets have apparent opposing roles, e.g. Ca^2+^/CaM-dependent kinase and Ca^2+^/CaM-dependent phosphatase. Therefore, it has been postulated that CaM can regulate its targets through its compartmentalization in the cell [25], [26]. For example, hormonal stimulation translocates CaM to the nucleus and enhances its activation of nuclear targets [27]. This model of redistribution between compartments, however, cannot explain CaM-mediated regulation of opposing targets within a small compartment such as the dendritic spine. Here, we propose that CaM ultrastructural localization within dendritic spines can be regulated by Ng and that this localization influences synaptic plasticity.

The binding of Ca^2+^ enhances the affinity of CaM to most of its target proteins. Ng, however, is an exception because its affinity for CaM decreases with increased Ca^2+^. This unique property of Ng has led to two opposing views on its function. One view postulates that Ng sequesters CaM and constrains Ca^2+^/CaM-regulated signaling [10], [11]. The other view suggests that Ng concentrates and targets CaM within the spine to facilitate LTP induction [6], [12], [13]. In the present study, we provide direct evidence that increasing Ng expression in CA1 hippocampal neurons causes CaM to translocate closer to the plasma membrane. We have previously shown that a significant fraction of Ng (31.3% of immunolabeling of endogenous Ng labeling) is present at the extrasynaptic plasma membrane next to the PSD, suggesting that Ng itself is targeted to the plasma membrane within dendritic spines [14]. Since the only known function of Ng is to bind CaM, overexpression of Ng within the spine causes more CaM to shift to where Ng is localized and as a result, CaM is translocated closer to the plasma membrane. In this study, we also show that Ng overexpression, which results in CaM targeting within the spine, is sufficient to lower LTP induction threshold. These results also provide an explanation of our previous study where overexpression of CaM, unlike Ng, was not able to enhance synaptic strength. Together, these results highlight the physiological relevance of CaM distribution within the spine.

There are quite a few CaM-regulated enzymes that are of interest for synaptic plasticity, e.g. adenylyl cyclases, calcineurin and protein kinases. CaMKII, however, has been of special interest because of its unique features. For example, CaMKII can act as a molecular switch, i.e. once activated by Ca^2+^-CaM, its activity can persist through its autophosphorylation even after the return of Ca^2+^ signal to baseline [28]. CaMKII is also required [29], [30] and sufficient [31] for LTP induction. Excellent reviews for the roles and mechanisms of CaMKII in LTP are available [32], [33], [34], [35]. Interestingly, there is a close correlation between Ng level and CaMKII activity [36], [37], [38], [39]. Nonetheless, while Ng knockout mice show decreased CaMKII activity compared to wild type [40], overexpression of Ng shows increased CaMKII activity in the synaptosomal fraction [14]. Our findings that CaM and CaMKII have a similar lateral, but not vertical, distribution within dendritic spines highlight the importance of their ultrastructural localization.

Analysis of CaM distribution within the spine reveals that there is a relatively high fraction of CaM at the PSD. This observation supports previous biochemical findings that identified CaM as a major component of the PSD [41], [42]. In contrast to CaM, only 5.7% of Ng immunolabeling was found at the PSD [14], suggesting that a large fraction of CaM at the PSD is not associated with Ng, and thus is unlikely to be involved in Ng-CaM-CaMKII signaling pathway. Such finding is not surprising given the plethora of targets that bind to CaM at the PSD [43], [44]. Thus, the fact that a fraction of the intraspine CaM is specifically translocated closer to the membrane, when Ng is overexpressed, highlights the relevance of this particular CaM pool in LTP induction.

Our data support a model in which Ng targets CaM closer to the plasma membrane within dendritic spines, where CaMKII is also more concentrated, and supports LTP through the preferential activation of CaMKII. CaM targeting within the spine is apparently essential for efficient CaMKII and LTP induction. For example, Ng knockout mice have impaired CaMKII activation and LTP induction [40]. Even acute knockdown of Ng resulted in failure of LTP induction [14]. Moreover, a computational study has supported a need for high CaM concentration within the spine to be able to activate CaMKII by short Ca^2+^ signals [13]. Accordingly, it is likely that a lack of Ng may lead to an inadequate level of CaM being targeted close to the plasma membrane, which subsequently would fail to fully activate CaMKII.

This model predicts that CaM targeting by Ng may regulate learning and memory. This notion is supported by Ng knockout studies, which showed that Ng knockout mice exhibit spatial memory deficits [11], [40]. Interestingly, studies with mice that are heterozygotes for Ng showed that there is a correlation between Ng levels and memory performance, where lower Ng levels correlated with lower memory performance [45]. Importantly, there are a number of neurological disorders, which are characterized by memory deficits, that are accompanied with decreased Ng levels. For example, Ng immunoreactivity was dramatically reduced in areas of prefrontal cortex in postmortem schizophrenia brain tissues [46]. As a result, a number of studies have investigated the potential involvement of Ng in schizophrenia [47], [48], [49]. A significant role of Ng in schizophrenia is strongly supported by a genome-wide scan of thousands of schizophrenia and control cases that identified Ng as one of four major variants associated with the disease [50]. Similarly, chromosomal microarray mapping suggests a role of Ng in cognitive deficits in Jacobsen syndrome [51]. Alzheimer’s disease is also accompanied by decreased Ng levels [52] and the Ng mRNA fails to be delivered to neuronal dendrites in Alzheimer’s disease, suggesting that Ng protein level is decreased locally at synapses in this disease [53]. An interesting question that arises from the current study is whether CaM targeting is disrupted in the before-mentioned diseases as well as in the Ng knockout mice. Our model predicts that CaM will not be enriched close to the plasma membrane in dendrtitic spines of affected neurons. Another interesting question that also warrants further testing is whether increasing Ng (and hence CaM targeting) will be sufficient to enhance learning and memory and/or capable of enhancing memory performance in animal models of the before-mentioned conditions.

An additional important question remains to be answered is how Ng itself is targeted to the plasma membrane. While, through its IQ motif, Ng can bind to CaM and target it closer to the plasma membrane, it is not well understood how Ng concentrates at the plasma membrane. *In vitro* study showed that Ng can bind to phosphatidic acid (PA) [54]. Thus, it is possible that through this interaction, Ng is targeted to the plasma membrane. Another potential mechanism by which Ng may bind and accumulate at the plasma membrane is through possible palmitoylation of two cysteine residues at its N-terminus. Interestingly, growth-associated protein 43 (GAP-43), another neural-specific CaM-binding protein expressed mainly presynaptically, is tethered to presynaptic membrane through two palmitoylated cysteine residues [55]. Finally, it is possible that Ng binds to an unidentified protein that targets it specifically at the spine plasma membrane. Future studies are needed to explore these possibilities.

In conclusion, our results provide insight into the significant physiological role of CaM targeting within the spine and introduces a novel mechanism by which LTP threshold is regulated.
